# Perivascular Epithelioid Cell Tumor with Uncertain Malignant Potential Arising in the Round Ligament

**DOI:** 10.3390/diagnostics14060616

**Published:** 2024-03-14

**Authors:** Alina Badlaeva, Anna Tregubova, Diana Kruglyak, Irina Luzhina, Aleksandra Asaturova

**Affiliations:** National Medical Research Center for Obstetrics, Gynecology and Perinatology Named after Academician V.I. Kulakov of the Ministry of Health of Russia, Bldg. 4, Oparina Street, Moscow 117513, Russia; a_badlaeva@oparina4.ru (A.B.); a_tregubova@oparina4.ru (A.T.); d_kruglyak@oparina4.ru (D.K.); i_lugina@oparina4.ru (I.L.)

**Keywords:** perivascular epithelioid cell tumor, broad ligament, uncertain malignant potential, melanocytic markers

## Abstract

A 12-year-old adolescent was diagnosed with a right-sided solid mass in the round ligament of the uterus. The chief complaints were abdominal pain and pelvic discomfort. She underwent laparoscopic tumor resection. Histological examination demonstrated a trabecular growth pattern of epithelioid cells with mitotic activity (3 per 50 HPF), which expressed melanocytic and myoid markers. Due to aforementioned findings, a final diagnosis of perivascular epithelioid cell tumor (PEComa) with uncertain malignant potential was made. To the best of our knowledge, this localization of PEComa is considered to be infrequent with only occasionally reported cases.

PEComas (perivascular epithelioid cell tumors) are a rare group of mesenchymal neoplasms consisting of perivascular cells with epithelioid morphology. Recent evidence suggests that these tumors derive from neural crest stem cells so PEComas show expression of both melanocytic and myoid markers [1,2]. PEComas could be localized in a variety of anatomical sites, e.g., the lung, kidney, uterus, and breast [3]. Due to PEComas’ clinical behavior, they may be classified into benign, uncertain malignant potential, and malignant tumors [3,4,5,6,7,8]. A special algorithm has been proposed for assessing the accurate diagnosis. Moreover, PEComas arising from the female genital tract should be stratified based on gynecology-specific criteria, such as tumor size > 5 cm, nuclear atypia, mitotic count > 1/50 high power fields (HPF), necrosis, and lymphovascular invasion [9]. A tumor with uncertain malignant potential must meet from one to two of the above-mentioned features. The presence of three or more features indicates a malignant PEComa [10,11].

According to many experts in this field, PEComas are seen at a high frequency in the tuberous sclerosis complex due to inactivating germline mutations TSC1/TSC2 (tuberous sclerosis complex 1/2) [12,13]. In addition, the latter could not only be germline, but also sporadic in the above-mentioned neoplasm [12]. The prior literature also highlights TFE3 (transcription factor E3)-rearranged PEComas with a lack of TSC1/2-inactivating mutations [12,13]. As a result, mTOR pathway contributes to tumorogenesis in PEComas, thereby giving an opportunity for target therapy with mTOR inhibitors [12,13,14,15].

**Figure 1:**
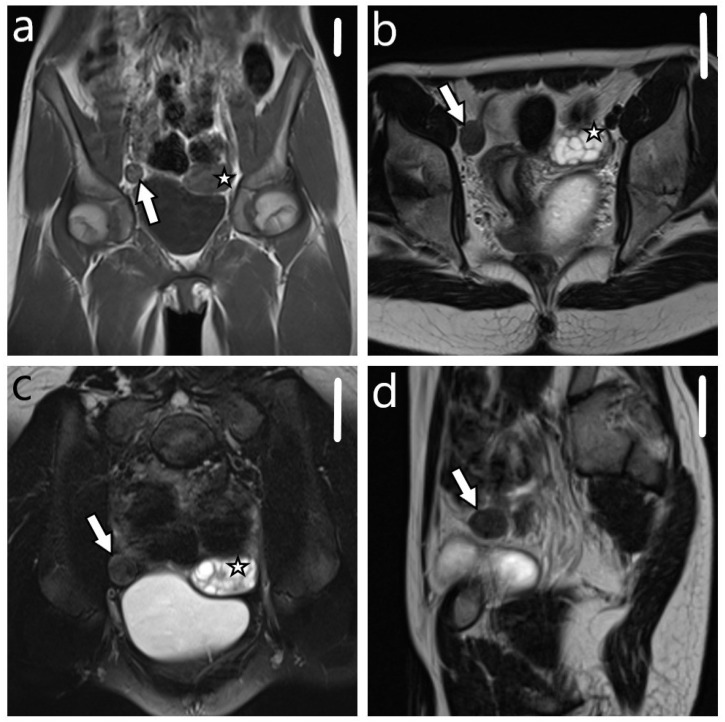
A 12-year-old adolescent who complained of abdominal distention and pelvic discomfort was admitted to the hospital. A magnetic resonance imaging (MRI) scan showed a large right-sided well-circumscribed pelvic solid mass arising in the round ligament measuring 2.5 × 2.0 × 2.0 cm (**a**–**d**, arrow; scale bar 5 cm). It has an *isointense* signal on T1WI ((**a**)—coronal plane), hypointense signal on T2WI ((**b**–**d**)—coronal, axial, and sagittal planes, respectively). There was a restricted diffusion on the DWI and ADC map. The initial MRI was interpreted as intraligamentary leiomyoma. The left ovary with multiple follicles was visualized (**a**–**c**, asterisk).

**Figure 2:**
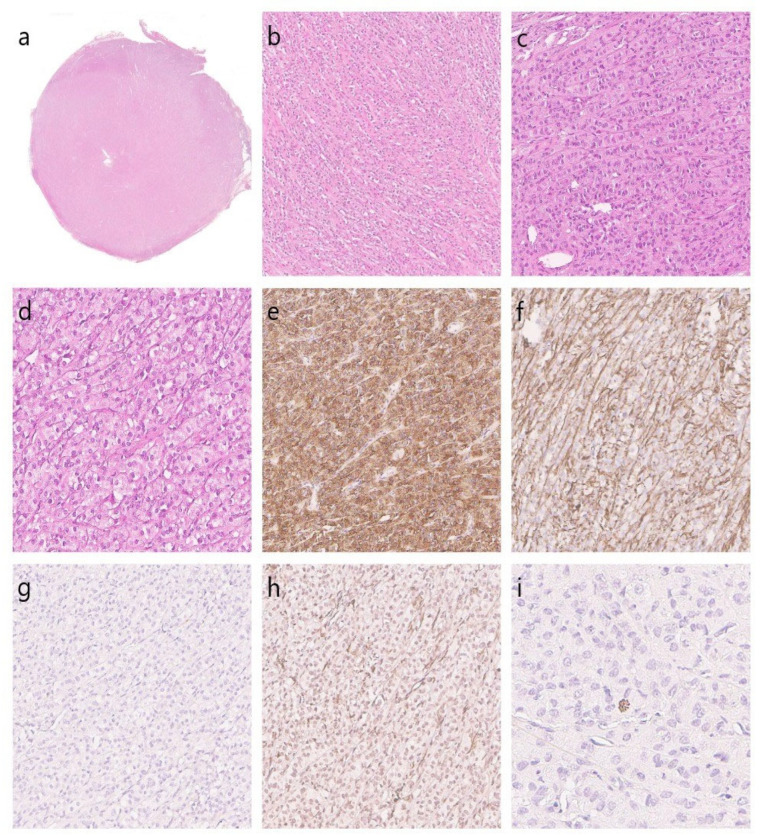
The patient was treated with laparoscopic resection of the right round ligament. Gross examination showed encapsulated a tan-pink friable mass. The 2.5 cm tumor was well-circumscribed ((**a**), **2×**). Microscopically, this neoplasm with distinctive borders predominantly exhibited trabecular and nested architectural patterns ((**b**), **20×**), without spindled morphology. It also displayed a network of small thick-walled vessels with tumor cells in perivascular arrangements ((**c**), **50×**). Necrosis, as well as lymphovascular invasion was not found. The neoplasm was composed of epithelioid medium cells with pale eosinophilic to clear cytoplasm and well-defined cytoplasmic borders ((**d**), **100×**). Cells had a centrally situated somewhat irregular nucleus. The latter was round to oval, uniform without significant atypia and pleomorphism. The mitotic rate was 3 mitotic figures per 50 HPF. Melanin-laden epithelioid cells were not found. Immunohistochemistry was performed with the following results. Tumor cells showed dot-like cytoplasmic immunoreactivity for melanocytic marker HMB45 ((**e**), **100×**), as well as Melan A. To a lesser extent, epithelioid cells stained for myogenic marker H-caldesmon ((**f**), **100×**). Tumor cells demonstrated the absence of immunostaining for inhibin α ((**g**), **50×**). The neoplastic tissue was also negative for actin, smooth muscle ((**h**), **50×**) and desmin. Mitotic figures were difficult to find so phosphohistone H3 (PHH3) antibody was used ((**i**), **200×**). Because of the revealed morphological feature (3 mitoses/50 HPF), we attributed the neoplasm to uncertain malignant potential.

In this case, the differential diagnosis included epithelioid intraligamentary leiomyoma and, less likely, GIST. The presence of perivascular arrangements of epithelioid cells as well as a distinctive immunophenotype supports the initial diagnosis of PEComa. Tumor resection was complete, with no margin involvement. The 3-month follow-up showed no recurrence using ultrasound examination.

## Data Availability

The authors declare that all the data described in this article are available upon reasonable request.
